# The Healthy Eating Index-2015 and All-Cause/Cause-Specific Mortality: A Systematic Review and Dose–Response Meta-Analysis

**DOI:** 10.1016/j.advnut.2023.100166

**Published:** 2024-02-23

**Authors:** Xuanyu Hao, Dongyang Li

**Affiliations:** 1 The Department of Gastroenterology at Shengjing Hospital of China Medical University, Shenyang, Liaoning, P.R. China; 2 The Department of Urology at Shengjing Hospital of China Medical University, Shenyang, Liaoning, P.R. China

**Keywords:** Healthy Eating Index-2015, all-cause mortality, cancer-cause mortality, CVD-cause mortality, dose–response, meta-analysis

## Abstract

This meta-analysis was undertaken to determine the predictive value of Healthy Eating Index (HEI)-2015 in all-cause, cancer-cause, and cardiovascular disease (CVD)-cause mortality. This review was registered with PROSPERO as CRD42023421585. PubMed and Web of Science were searched for articles published by September 15, 2023. The hazard ratio (HR) was calculated with exact confidence intervals (CIs) of 95%. Statistical heterogeneity among studies was measured by Cochran's *Q* test (*χ*^2^) and the *I*^2^ statistic. Eighteen published studies were finally identified in this meta-analysis. The results showed that the HEI-2015 was associated with all-cause mortality either as a categorical variable (HR: 0.80; 95% CI: 0.79, 0.82) or continuous variable (HR: 0.90; 95% CI: 0.88, 0.92). The HEI-2015 was also associated with cancer-cause mortality as categorical variable (HR: 0.81; 95% CI: 0.78, 0.83) or continuous variable (HR:  0.90; 95% CI: 0.81, 0.99). The categorical HEI-2015 was also independently correlated with decreasing CVD-cause mortality (HR: 0.81; 95% CI: 0.75, 0.87). A nonlinear dose–response relation between the HEI-2015 and all-cause mortality was found. In the linear dose–response analysis, the risk of mortality from cancer decreased by 0.42% per 1 score increment of the HEI-2015 and the risk of CVD-cause mortality decreased by 0.51% with the increment of the HEI-2015 per 1 score. Our analysis indicated a significant relationship between the HEI-2015 and all-cause, cancer-cause, and CVD-cause mortality.

**Statement of Significance**

This is the first meta-analysis to describe the pooled risk of mortality from all-cause, cardiovascular disease, and cancer according to dietary patterns defined by the Healthy Eating Index-2015.

## Introduction

Diet plays an important role in human health and the process of growth. Poor diet quality and the imbalanced dietary intake have been suggested to be a significant risk factor for adverse health outcomes and accounts for disease morbidity, even mortality [1]. Instead of playing a separate role, food components in one dietary pattern may have the possible synergistic or interactive effects among each other [2]. The importance of dietary patterns to consider foods and nutrients in combination has been emphasized over analyzing the individual nutrient or food components [3]. Moreover, through integrating the isolated nutrients, the eating patterns could reflect the real-world dietary practices more precisely and provide the more reliable evidence for dietary recommendations [4].

On the basis of evidence-based analysis, dietary indices play an important role in assessing dietary patterns [5]. As a commonly used diet-quality indicator, the Healthy Eating Index (HEI), which was created to reflect adherence to the dietary pattern recommended by the Dietary Guidelines for Americans (DGA), is periodically updated on the basis of the most recently released DGA to evaluate dietary quality [6]. Regarding the healthy eating patterns comprised with the different nutrients or foods as a whole, the HEI-2015 contains 13 dietary components, including 9 adequacy components (including total fruits, whole fruits, total vegetables, greens and beans, whole grains, dairy, total protein foods, seafood, plant proteins and fatty acids), and 4 moderation components (including refined grains, sodium, added sugars, and saturated fats) [7]. The maximum value of each component is designed to be scored from 5 to 10 and the full range of the HEI-2015 is 0–100. Greater adherence to a healthy dietary pattern, such as the Mediterranean diet, can reduce the incidence of noncommunicable diseases (NCDs) and bring a positive influence on health [8]. NCDs, including cardiovascular disease (CVD), diabetes, metabolic syndrome (MetS), and cancer, contribute to the risk of death as a leading cause in the world [9]. Previous studies have revealed the inverse association between the scores of the HEI-2015 and the risk of various cancers, including breast cancer, oral and pharyngeal cancer, as well as lung cancer [[10], [11], [12]]. In addition, higher diet quality measured by the HEI-2015 has been shown to be associated with the lower risk of CVD, diabetes and MetS [4,13,14].

Up to now, multiple studies have shown the correlation between mortality incidence and the HEI-2015, but no dose–response meta-analysis has been performed [[15], [16], [17], [18], [19], [20], [21], [22], [23], [24], [25], [26], [27], [28], [29], [30], [31], [32], [33], [34]]. To provide a more comprehensive and detailed assessment of the relationship between the scores of the HEI-2015 and mortality, we conducted this systematic review and meta-analysis of published studies to investigate whether the HEI-2015 is associated with mortality from CVD, cancer, and all-cause mortality and to determine the shape of the dose–response relationship for the first time.

## Materials and Methods

### Search strategy

This meta-analysis was performed in accordance with the recommendations of the PRISMA statement (Supplemental Table 1). This review was registered with PROSPERO as CRD42023421585. PubMed and Web of Science were searched from inception through September 15, 2023. The main search items were as follows: (“HEI-2015” OR “Healthy Eating Index-2015”) AND (“mortality” OR “mortalities” OR “death” OR “survival” OR “prognosis” OR “fatal” OR “survive”). No restrictions were imposed on the languages of publications. A manual search of the reference lists from all the related studies including review articles was performed to identify the additional relevant publications as well. Two researchers (XYH and DYL) assessed the study eligibility and obtained the full articles of the potentially relevant studies for detailed evaluation independently, and any inconsistencies were resolved by consensus.

### Inclusion and exclusion criteria

The original articles were considered to be eligible if they met the following criteria: *1*) cohort studies (prospective or retrospective) or cross-sectional studies, *2*) human study, *3*) the study investigated the association between the HEI-2015 and risk of mortality, and *4*) the effect estimates including hazard ratio (HR) and 95% confidence intervals (CIs) obtained from the original data or the effect estimates available to be calculated. The exclusion criteria were as follows: *1*) data were not available or abstract only, *2*) animal studies, case reports, commentary articles, experimental studies, or letters to editors, and *3*) duplicated studies.

### Risk of bias

The risk of bias in the included studies was appraised using the Risk of Bias in Non-randomized Studies – of Exposures (ROBINS-E) tool by 2 authors (XYH and DYL) independently [35]. Seven domains of bias were covered, and for each domain, the risk of bias was graded as low, moderate, serious, or critical. An overall risk of bias of included studies was also provided. Any discrepancy was discussed by the 2 authors.

### Data extraction

The following data were extracted after the full-text articles were reviewed: the first author’s name, publication year, study location, duration of follow-up, age, sample size, outcomes (all-cause, CVD-cause, and cancer-cause mortality), number of deaths, proportion of female participants, effect estimates and their 95% CIs (only estimates adjusted for the covariates indicated in Table 1 were included), multivariate adjustments, and the risk of bias for each article. Two authors (XYH and DYL) independently extracted data from the articles, any disagreements were resolved by discussion.

**TABLE 1:** Characteristics of studies included in the meta-analysis

Reference	Study	Data sources	Participants	Country	Follow-up (y)	Age at baseline (y)	Exposure assessment	Study size		Outcome assessment				Adjustments	Sources of funding
						(mean ± SD or range)		Total participants (*n*)	Female proportion (%)	Number of death (*n*)	Cause of death	Type of data and comparison	Pooled HR (95% CI)		
[15]	Myneni et al. 2021	WHI OS	Postmenopausal women	United States	17.3	50–79	122-item FFQ	86,090	100	1393	Cancer-cause	Categorical Quintile 5 vs. Quintile 1	0.86 (0.72, 1.04)	Age, race, education, BMI, physical activity, active smoking, years of exposure to secondhand smoke during childhood and as an adult, and energy intake.=	No information
[16]	Panizza et al. 2018	MEC	Multiethnic populations	United States	17–22	45–75	Quantitative FFQ	156,804	55.25	51,442	All-cause	Categorical Quintile 5 vs. Quintile 1	0.79 (0.76, 0.82)	Age at study entry, BMI, history of diabetes, energy, ethnicity, education, marital status, smoking, weekly hours of moderate to vigorous physical activity, and alcohol intake	NCI at the NIH
											CVD-cause		0.76 (0.71, 0.82) for men 0.75 (0.7, 0.81) for women		
											Cancer-cause		0.8 (0.75, 0.87) for men 0.84 (0.78, 0.91) for women		
[17]	Haslam et al. 2023	BCFR	Women diagnosed with a first primary, invasive breast cancer	United States and Canada	11.3	52.8 ± 23.5	108-item FFQ	6157	100	1265	All-cause	Categorical Quartile 4 vs. Quartile 1	0.88 (0.74, 1.04)	Age, study site, total caloric intake, race and ethnicity, education, treatment type, tumor stage, recent recreational physical activity, cigarette smoking status, and pack-years of cigarette smoking, tumor estrogen receptor status, tumor progesterone receptor status, and menopausal status	NCI and NIH
[18]	Hu et al. 2019	ARIC study	Participants from 4 United States communities	United States	24–25	45–64	66-item FFQ	12,413	56	1722	All-cause	Categorical Quintile 5 vs. Quintile 1	0.82 (0.75, 0.89)	Age, sex, race center, total energy intake, education level, income level, physical activity, smoking status, alcohol status	NHLBI, NIH, the Department of Health and Human Services, NIDDK
										5747	CVD-cause		0.68 (0.58, 0.8)		
[19]	Hu et al. 2020	CRIC study	People with CKD	United States	12	21–74	124-items DHQ	2403	48	773	All-cause	Categorical Tertile 3 vs. Tertile 1	0.76 (0.63, 0.92)	Total energy intake, clinical site, age, sex, race, education, income level, estimated glomerular filtration rate, urinary protein, smoking status, physical activity, and alcohol status, BMI, diabetes mellitus, hypertension, cardiovascular disease, high-density lipoprotein cholesterol, and angiotensin-converting enzyme inhibitor or angiotensin II receptor blocker use	NIDDK, Perelman School of Medicine at the University of Pennsylvania CTSA NIH/NCATS, Johns Hopkins University, University of Maryland, the Clinical and Translational Science Collaborative of Cleveland, NCATS component of the NIH and NIH Roadmap for Medical Research, Michigan Institute for Clinical and Health Research, University of Illinois at Chicago CTSA, Tulane Center of Biomedical Research Excellence for Clinical and Translational Research in Cardiometabolic Diseases, Kaiser Permanente NIH/National Center for Research Resources University of California San Francisco-Clinical & Translational Science Institute, NHLBI, National Institute of General Medical Sciences
[20]	Wang et al. 2020	SBCSS	Women with a primary breast cancer diagnosis	China	5	25–70	FFQ	3450	100	374	All-cause	Categorical Quartile 4 vs. Quartile1	0.79 (0.57, 1.1)	Age, interval between diagnosis and dietary survey, total energy intake, income, education, marriage, menopausal status at diagnosis, BMI at 60-mo survey, physical activity at 60-mo survey, ER, PR, HER2, TNM stages, comorbidity, chemotherapy, radiotherapy and immunotherapy	United States Department of Defense Breast Cancer Research Program, NCI at the NIH, National Natural Science Foundation of China
												Continuous HEI-2015	0.94 (0.85, 1.03)		
[21]	Reedy et al. 2018	NIH-AARP Diet and Health Study	American retired participants	United States	15–16	50–71	124-item FFQ	422,928	43%	53,826 for men 30,948 for women	All-cause	Categorical Quintile 5 vs. Quintile 1	0.8 (0.78, 0.82) for men 0.77 (0.74, 0.8) for women	Age, race/ethnicity, education, marital status, physical activity, smoking, energy, BMI, diabetes, alcohol	Canadian Cancer Society Research Institute Capacity Development Award, NCI Cancer Center Support Grant, Comprehensive Cancer Center of Wake Forest Baptist Medical Center
											CVD-cause		0.87 (0.83, 0.92) for men 0.79 (0.73, 0.85) for women		
											Cancer-cause		0.78 (0.74, 0.82) for men 0.8 (0.75, 0.86) for women		
[22]	Chebet et al. 2020	WHI	Postmenopausal black women	United States	13	50–79	FFQ	9886	100	313	Obesity-related cancer-cause	Continuous HEI-2015	0.98 (0.85, 1.12)	Age, BMI, waist circumference, smoking, educational attainment, income, randomization WHI arm, participating in observation study, and sedentary time	NCI at the NIH, NHLBI, NIH, United States Department of Health and Human Services
											Breast cancer-cause		0.96 (0.77, 1.22)		
											Colorectal cancer-cause		1.02 (0.74, 1.41)		
[23]	Jayanama et al. 2021	NHANES (2007–2012)	United States population	United States	8	47.2 ± 16.7	24-h dietary recall interviews	15,249	51.7	1171	All-cause	Continuous HEI-2015	0.92 (0.88, 0.96)	Age, sex, race, educational level, marital status, employment status, smoking, study cohort, and BMI	None
[24]	Ha et al. 2020	NHANES(1988-1994, 1999–2006)	United States population	United States	9–27	≥30	24-h dietary recall interviews	23,797	51.2	8106	All-cause	Categorical Quintile 5 vs. Quintile 1	0.87 (0.77, 0.98)	Age, gender, race/ethnicity, BMI, and total energy intake, poverty-income ratio, marital status, physical activity, history of cardiovascular diseases, history of diabetes, and history of hypertension, smoking status	Basic Science Research Program through the NRF funded by the Ministry of Education
[25]	Li et al. 2023	NHANES (1999–2014)	United States population	United States	15	51.7	24-h dietary recall interviews	8363	48.1	991	All-cause	Categorical Quartile 4 vs. Quartile 1	0.78 (0.58, 1.05)	Age, sex, and race/ethnicity, education level, family income-to-poverty ratio, marital status, smoking status, drinking status, physical activity, BMI, total energy intake, and history of hypertension, hypercholesterolemia, or CVD	National Nature Science Foundation of China, the Hubei Province Science Fund for Distinguished Young Scholars, National NutritionScience Research Grant, the Fundamental Research Funds for the Central Universities, the China Postdoctoral Science Foundation
												Continuous HEI-2015	0.86 (0.74, 1)		
[26]	Luo et al. 2020	Guangdong Liver Cancer Cohort	Patients with newly diagnosed and previously untreated HCC	China	2.1	51.9 ± 12.0	79-item FFQ	887	11.3	389	All-cause	Categorical Tertile 3 vs. Tertile 1	0.86 (0.67, 1.11)	Age at diagnosis, sex, energy intake, BMI, smoking status and education level, alcohol drinking status, C-reactive protein level, alpha-fetoprotein level, Child–Pugh class, TNM stage and cancer treatment	Natural Science Foundation of China, the Natural Science Foundation of Guangdong Province, China
												Continuous HEI-2015	0.86 (0.74, 1.01)		
										347	HCC-cancer	Categorical Tertile 3 vs. Tertile 1	0.93 (0.71, 1.21)		
												Continuous HEI-2015	0.91 (0.78, 1.07)		
[27]	Hashemian et al. 2019	Golestan Cohort Study	Iran population	Iran	10.6	52.1 ± 8.9	116-item FFQ	42,373	57.7	4424	All-cause	Categorical Quintile 5 vs. Quintile 1	0.92 (0.83, 1.01)	Age, sex, BMI, formal education, place of residence, smoking status, opium use, physical activity, wealth score, marital status, history of hypertension, and total energy intake	United States. Department of Health and Human Services, Tehran University of Medical Sciences ,Cancer Research United Kingdom, the Intramural Research Program of the United States ,NCI,NIH, the International Agency for Research on Cancer
											CVD-cause		1 (0.86, 1.17)		
											Cancer-cause		0.79 (0.64, 0.98)		
[28]	Lopez-Pentecost et al. 2022	WHI	Postmenopausal Hispanic women	United States	11.9	60.1 ± 6.7 for non-cancer participants, 61.0 ± 6.7 for cancer participants	122-item FFQ	5482	100	220	Cancer-cause	Categorical Tertile 3 vs. Tertile 1	0.9 (0.6,1.33)	Age, language, education, cancer family history in a first relative, cancer screening, recreational physical activity, and study arm	PA-16-288 Research Supplement to Promote Diversity in Health-Related Research
[29]	Gicevic et al. 2021	NHANES(2003–2008)	United States population	United States	9.2	58 ± 13	Two 24-h dietary recall interviews	5525	52	767	All-cause	Categorical Quintile 5 vs. Quintile 1	0.77 (0.57, 1.03)	Age, sex, race/ethnicity, day of week, smoking status, alcohol use, physical activity, BMI	IMMANA Postdoctoral Fellowship Research Grant (SG)
											Cancer-cause	Continuous HEI-2015	0.92 (0.84, 1.01)		
[30]	Park et al. 2022	MEC	Multiethnic populations	United States	10	45–75	Quantitative FFQ	70,045	54.4	23,947	All-cause	Categorical Quartile 4 vs. Quartile 1	0.74 (0.67, 0.82) in cancer survivors 0.84 (0.8, 0.87) for participants without cancer at first	Sex, age, race/ethnicity, education, BMI, physical activity, marital status, comorbidity, total energy intake, menopausal hormone therapy for women, smoking status, average number of cigarettes, squared average number of cigarettes, number of years smoked, number of years since quitting, interactions between ethnicity and smoking status, average number of cigarettes, squared average number of cigarettes and number of years smoked, alcohol intake	NCI at the NIH
											Cancer-cause		0.84 (0.71, 1) in cancer survivors 0.85 (0.78, 0.93) for participants without cancer at first		
[31]	George et al. 2020	WHI OS	Postmenopausal women	United States	18.2	50–79	FFQ	59,388	100	9679	All-cause	Categorical Quintile 5 vs. Quintile 1	0.82 (0.76, 0.87)	Age in 5-y categories, BMI, race/ethnicity, and smoking, energy, alcohol, education, income, marital status, physical activity, and postmenopausal hormone replacement	Office of Disease Prevention, Office of the Director, National Institutes of Health, NHLBI, NIH, United States Department of Health and Human Services
											CVD-cause		0.94 (0.82, 1.08)		
											Cancer-cause		0.79 (0.7, 0.88)		
[32]	Shan et al. 2023	NHS and HPFS	Initially healthy women and men	United States	34 for women 36 for men	53.3 ± 9.6 for women 50.2 ± 7.2 for men	FFQ	119,315	36.9	22,900 for women 31,263 for men	All-cause	Categorical Quintile 5 vs. Quintile 1	0.81 (0.79, 0.84)	Age, calendar year, race and ethnicity, marriage status, living status, family history of myocardial infarction, family history of diabetes, family history of cancer, menopausal status, multivitamin use, aspirin use, total energy intake, smoking status, alcohol drinking, physical activity, history of hypertension, history of hypercholesterolemia, and BMI	NHLBI, NIH, NHLBI, NIDDK, the American Heart Association
												Continuous HEI-2015	0.88 (0.84, 0.91)		
											CVD-cause		0.87 (0.83, 0.92)		
											Cancer-cause		0.82 (0.78, 0.86)		
[33]	Li Fang et al. 2023	NHANES(2005–2018)	Hypertension patients from United States population	United States	6.9	53.45 ± 16.05	24-h dietary recall interviews	27,618	46.96	3462	All-cause	Categorical Tertile 3 vs. Tertile 1	0.91 (0.76, 1.08)	Age, race, education level, marital status, PIR, physical activity, smoking, total energy intake (for all-cause mortality), eGFR, drinking, BMI (for all-cause mortality), DM, CVD (for all-cause mortality), dyslipidemia, cancer, anemia treatment, gout, COPD, HB, dialysis, CRP, and hypothyroidism	No information
										1064	CVD-cause		0.88 (0.61, 1.26)		
[34]	Wang et al. 2023	NHANES(1999–2008)	United States population	United States	11.2 ± 3.2	No information	24-h dietary recall interviews	11,939	No information	1149	All-cause	Categorical Tertile 3 vs. Tertile 1	0.87 (0.70, 1.08)	Sex, age, race, marital status, education, family poverty to income ratio, PAL, smoking, alcohol intake, CVD, cancer, diabetes, arthritis, cholesterol, hypertension, BMI	An Australia Awards Scholarship from The Department of Foreign Affairs and Trade, The Government of Australia, National Health and Medical Research Council of Australia
										222	CVD-cause		0.69 (0.47, 1.02)		
										263	Cancer-cause		0.77 (0.48, 1.26)		

Abbreviations: AARP, American Association of Retired Persons; ARIC, Atherosclerosis Risk in Communities; BCFR, Breast Cancer Family Registry; CKD, chronic kidney disease; CRIC, Chronic Renal Insufficiency Cohort; CTSA, Clinical and Translational Science Award; CVD, cardiovascular disease; DHQ, Diet History Questionnaire; FFQ, food frequency questionnaires; HCC, hepatocellular carcinoma; HEI, Healthy Eating Index; HPFS, Health Professionals Follow-up Study; MEC, Multiethnic Cohort Study; MET, metabolic equivalent; NCATS, National Center for Advancing Translational Sciences; NCI, National Cancer Institute; NHLBI, National Heart, Lung, and Blood Institute; NHS, Nurses’ Health Study; NIDDK, National Institute of Diabetes and Digestive and Kidney Diseases; NRF, National Research Foundation of Korea; SBCSS, Shanghai Breast Cancer Survival Study; WHI OS, Women’s Health Initiative Observational Study.

### Statistical analysis

Statistical analyses were conducted by using the STATA 12.0 software (Stata Corporation) and *P* < 0.05 was considered to be statistically significant. HR was calculated with exact 95% CIs. The HEI-2015 was applied as a continuous variable or categorical variable. Categories of HEI-2015 were based on tertiles, quartiles, and quintiles in different included studies. To reduce errors, the highest category was compared with the lowest category, and the lowest one was considered as the reference group. Statistical heterogeneity among studies was measured by Cochrane *Q* test (*χ*^2^) and *I*^2^ statistic: The *Q* test was used to test heterogeneity, and the *I*^2^ statistic was used to quantify the inconsistency. *P* < 0.1 for the *Q* test and *I*^2^ > 50% for the *I*^2^ statistic indicated that heterogeneity might exist across the studies. Therefore, a random effect model was used to pool the data; otherwise, a fixed effect model was adopted by utilizing the Mantel–Haenszel method. Subgroup assessments were performed according to gender (male or female), region (United States or Non-United States). Furthermore, to explore the sources of heterogeneity, we also conducted subgroup analyses on follow-up period and sample size, the values approaching median were selected as the cut-off points (15 y for follow-up period and 15,000 for sample size). Sensitivity was assessed to judge the reliability of the evidence. Meanwhile, funnel plot and Egger’s test were performed for evaluating publication bias.

The nonlinear dose–response meta-analyses were conducted to evaluate the relationship between the HEI-2015 and all-cause, cancer-cause, and CVD-cause mortality with the random-effects 4-knot cubic spline model in the STATA software [36]. The HR with the 95% CIs was extracted for each category of the HEI-2015, as well as data on the dose values of each HEI-2015 category, which were assigned with the midpoint for each category if they were unavailable. The person-years were also extracted from each study, which were calculated from cases and corresponding HRs if they were not given directly [37]. When the highest category was open-ended, the width was considered to be the same as the adjacent category interval. If the lowest category was open-ended, the lower bound was set to 0. To evaluate the difference between the nonlinear and linear models, the likelihood ratio test was used to test for nonlinearity. A linear dose–response analysis between the HEI-2015 and mortality was assessed with the method described by Greenland and Longnecker [38] to estimate the study specific slope lines.

## Results

### Literature search

**FIGURE 1:**
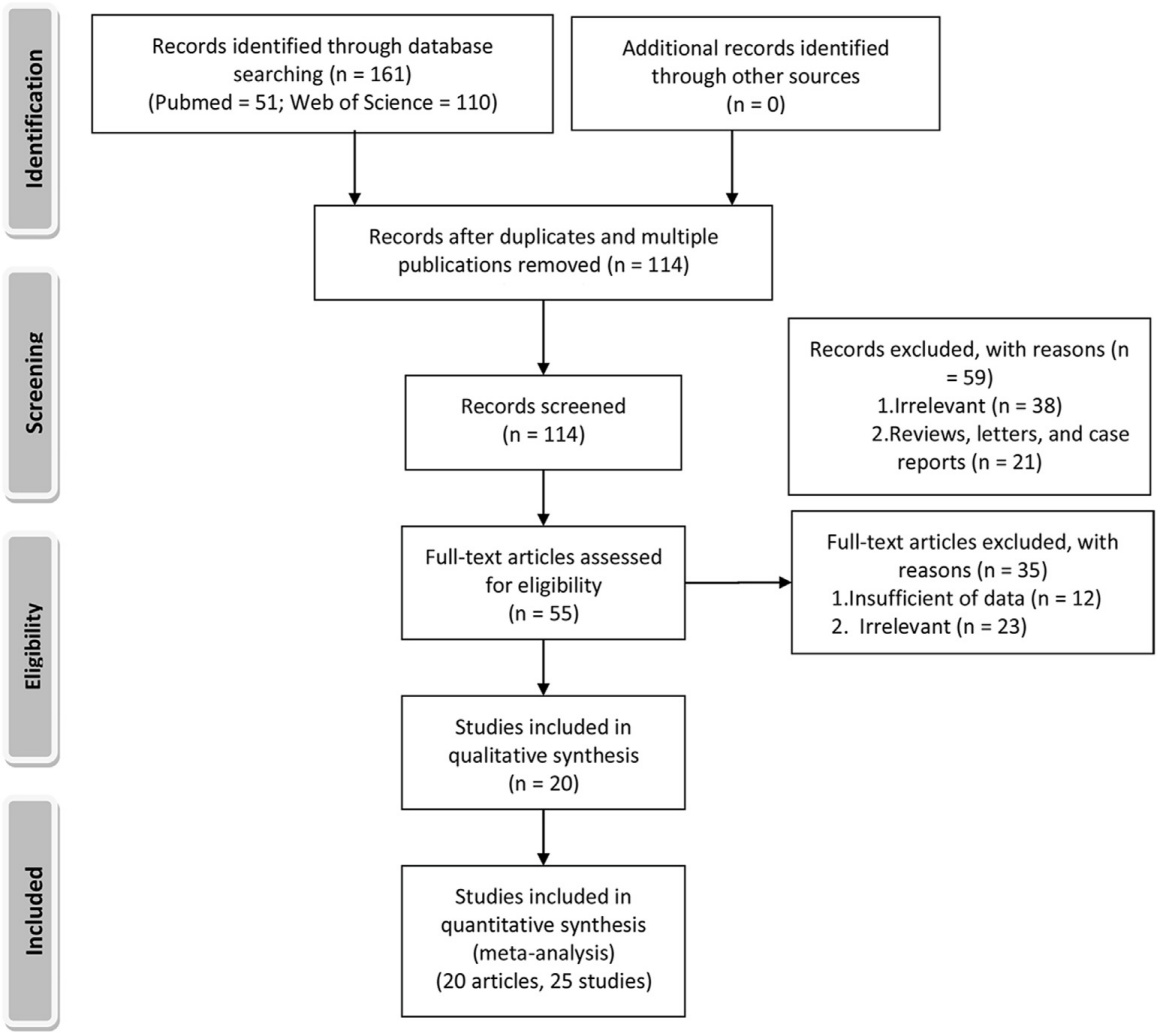
Flowchart of literature search and study selection.

A flowchart diagram of the article selection process is shown in Figure 1. A total of 138 records were obtained after searching for PubMed (*n* = 51) and Web of Science (*n* = 110). Among them, 59 articles were excluded after reviewing the title and abstract and 35 articles were excluded based on full-text reviewing. Finally, 20 articles and 25 studies were included in the meta-analysis [[15], [16], [17], [18], [19], [20], [21], [22], [23], [24], [25], [26], [27], [28], [29], [30], [31], [32], [33], [34]].

### Characteristics of included studies and risk of bias

A total of 20 published articles published from 2018 to 2023 were finally included in this meta-analysis. All included studies were prospective cohort studies. The characteristics of each study are presented in Table 1. A total of 1,065,175 study participants were involved in the analysis, whereas the duration of follow-up ranged from 797 d to 36 y. Among the included articles, 4 articles provided results reported males and females, as well as different kinds of mortality as outcomes independently, which were regarded as separate reports [16,21,22,30]. Geographically, 3 studies were conducted in Asia, 17 in North America. Among the 20 included studies, 8 studies reported CVD-mortality as outcome, 12 studies reported cancer-mortality as outcome, and 17 studies reported all-cause mortality as outcome.

Supplemental Table 2 presents the risk of bias for included studies evaluated by ROBINS-E tool. All included studies were judged to have high overall risk of bias. For risk of bias because of confounding, 16 studies [[15], [16], [17], [18],[20], [21], [22], [23], [24],[26], [27], [28], [29],[32], [33], [34]] were evaluated as high risk for the lack of important confounders and 4 studies [19,25,30,31] were assessed as some concerns because some confounding factors were based on self-reported measurements, which lower the validity and reliability. HEI-2015 were based on questionnaire or interviews in all studies that might lead to the recall bias; thus, all studies were considered at high risk of bias arising from measurement of the exposure. Twelve studies were assessed as some concern risk of bias because of missing data [15,17,[20], [21], [22], [23],^26^,[28], [29], [30], [31], [32]]. Separate predefined analysis plans were not available for any of the studies, it was indicated that the main analyses used for this meta-analysis were the primary objective of the study, so all studies were considered as some concern risk of bias in selection of the reported result.

### The HEI-2015 and all-cause mortality

**FIGURE 2:**
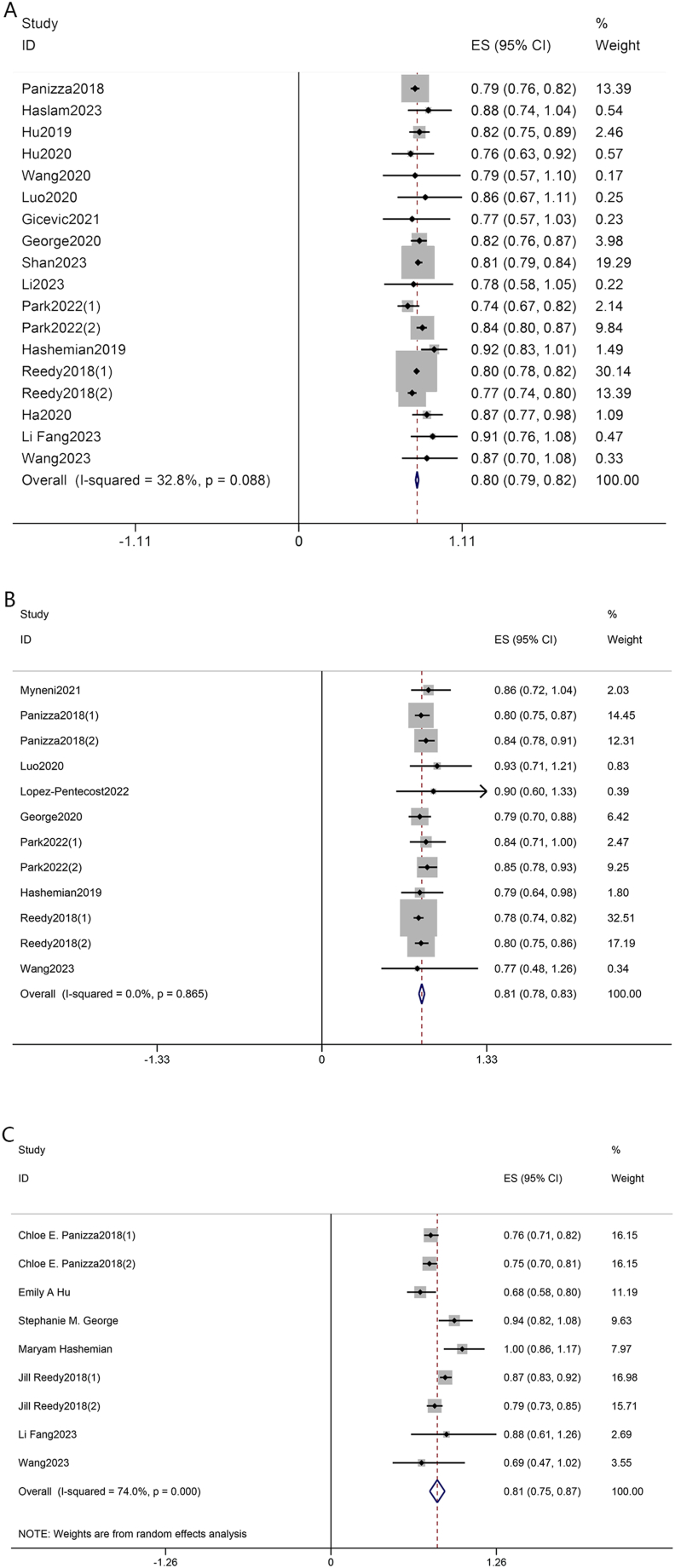
Forest plots of pooled HRs with 95% CI for HEI-2015 (the highest category compared with the lowest category) and all-cause mortality (A), cancer-cause mortality (B), and CVD-cause mortality (C). CI, confidence interval; CVD, cardiovascular disease; HEI, Healthy Eating Index; HR, hazard ratio.

**FIGURE 3:**
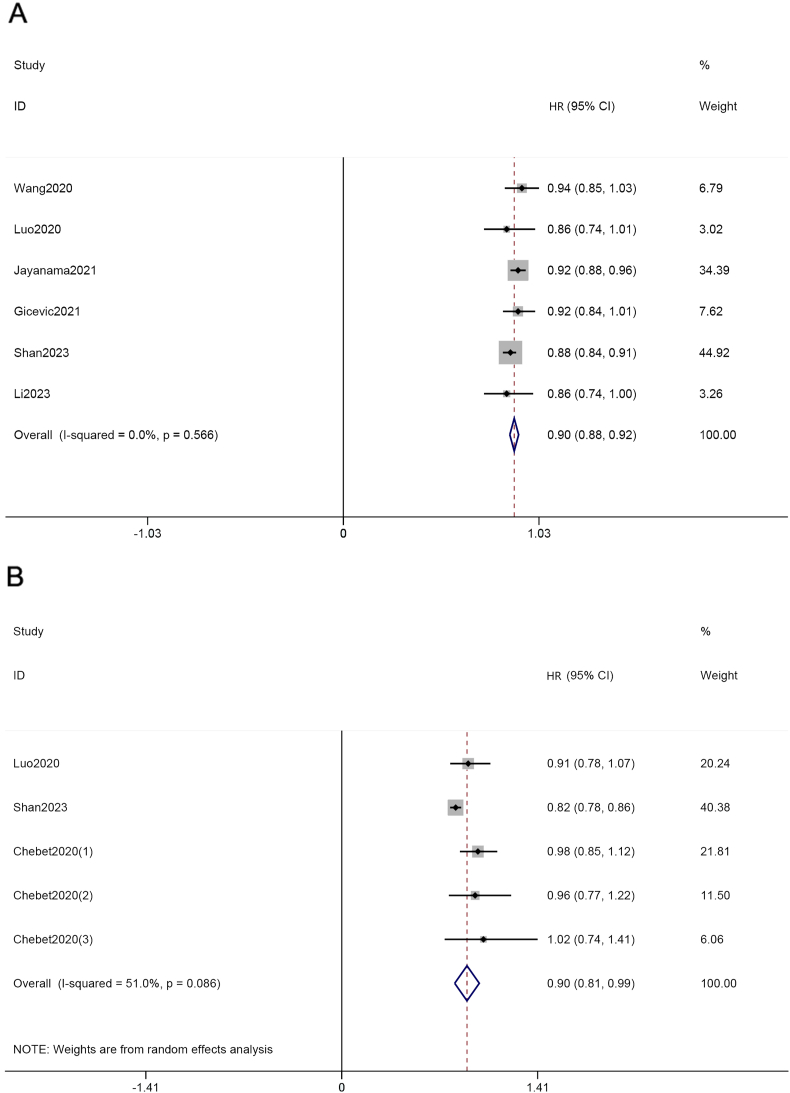
Forest plots of pooled HRs with 95% CI for HEI-2015 (continuous) and all-cause mortality (A), and cancer-cause mortality (B). CI, confidence interval; HEI, Healthy Eating Index; HR, hazard ratio.

**FIGURE 4:**
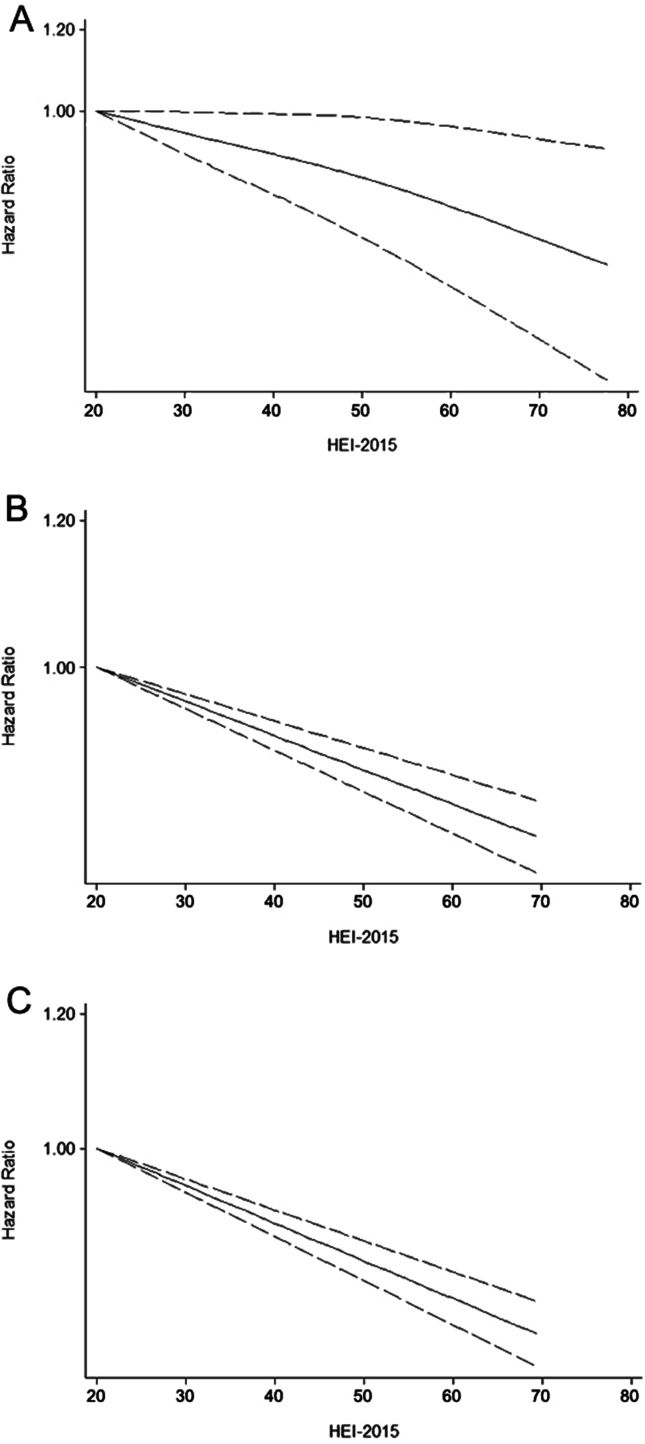
Dose–response analysis plots of HEI-2015 and (A) all-cause mortality, (B) cancer-cause mortality, and (C) CVD-cause mortality. CVD, cardiovascular disease; HEI, Healthy Eating Index.

As illustrated in Figure 2A, the results confirmed a significant association between HEI-2015 score and risk of all-cause mortality by the comparison of the highest and the lowest category of HEI-2015. Compared with the lowest category, the higher HEI-2015 had the pooled HR of 0.80 (95% CI: 0.79, 0.82) with a random model because the heterogeneity might exist (*I*^2^: 32.8%; *P* = 0.088). In addition, when regarded as a continuous variable, increased HEI-2015 was also independently related to a decreased all-cause mortality with the pooled HR of 0.90 (95% CI: 0.88, 0.92) calculated by a fix model because the heterogeneity was not statistically significant (*I*^2^: 0%; *P* = 0.566; Figure 3A). The dose–response analysis for the HEI-2015 and all-cause mortality is shown in Figure 4A, revealing a nonlinear association between the HEI-2015 and all-cause mortality, which showed a downtrend of all-cause mortality with the increment of the HEI-2015 (*P* for nonlinear = 0.0137).

### The HEI-2015 and cancer-cause mortality

The analysis revealed an association between HEI-2015 and cancer-cause mortality. Specifically, the highest category of HEI-2015 was associated with a lower risk of cancer-cause mortality compared with the lowest category of HEI-2015 (HR: 0.81; 95% CI: 0.78, 0.83), which was calculated by a fixed model because of the absence of heterogeneity (*I*^2^: 0%; *P* = 0.865) as presented in Figure 2B. We detected the relationship between the HEI-2015 and cancer-cause mortality when regarding the HEI-2015 as a continuous variable (HR:  0.90; 95% CI: 0.81, 0.99; *I*^2^: 51.0%; *P* = 0.086) and the result is shown in Figure 3B. In the dose–response analysis, the results of linear analysis illustrated that the risk of mortality from cancer decreased 0.42% per 1 score increment of the HEI-2015, as presented in Figure 4B (HR: 0.9958; 95% CI: 0.9949, 0.9967; *P* < 0.001), and a significant nonlinear dose–response relationship between the mortality risk from cancer and the HEI-2015 scores was not found (*P* for nonlinear = 0.1521).

### The HEI-2015 and CVD-cause mortality

The overall HRs estimated for the study participants in the highest category compared with the subjects in the lowest category of the HEI-2015 were compared with determine total risk estimates by utilizing a random effects model (HR: 0.81; 95% CI: 0.75, 0.87) with high heterogeneity observed across studies included (*I*^2^: 74%; *P* < 0.1), suggesting that the increased HEI-2015 might be associated with a lower incidence of CVD-cause mortality (Figure 2C). The linear dose–response analysis is shown in Figure 4C and the risk of CVD-cause mortality decreased by ∼0.51% with the increment of the HEI-2015 per 1 score (HR: 0.99 49; 95% CI: 0.9940, 0.9958; *P* < 0.001). The significant nonlinear association was not found between the HEI-2015 and CVD-cause mortality (*P* for nonlinear = 0.3433).

### Subgroup analysis and sensitivity analysis

**FIGURE 5:**
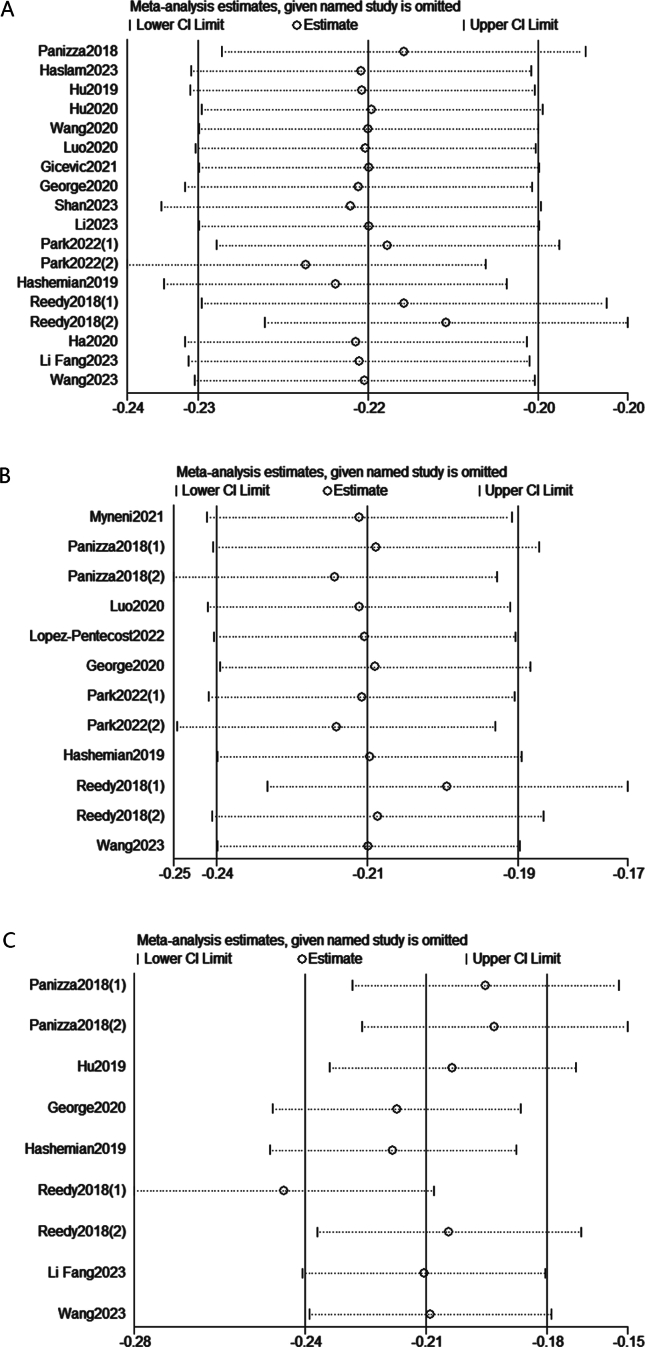
Sensitivity analysis of included studies for the highest HEI-2015 vs. the lowest category HEI-2015. (A) All-cause mortality. (B) Cancer-cause mortality. (C) CVD-cause mortality. CVD, cardiovascular disease; HEI, Healthy Eating Index.

**FIGURE 6:**
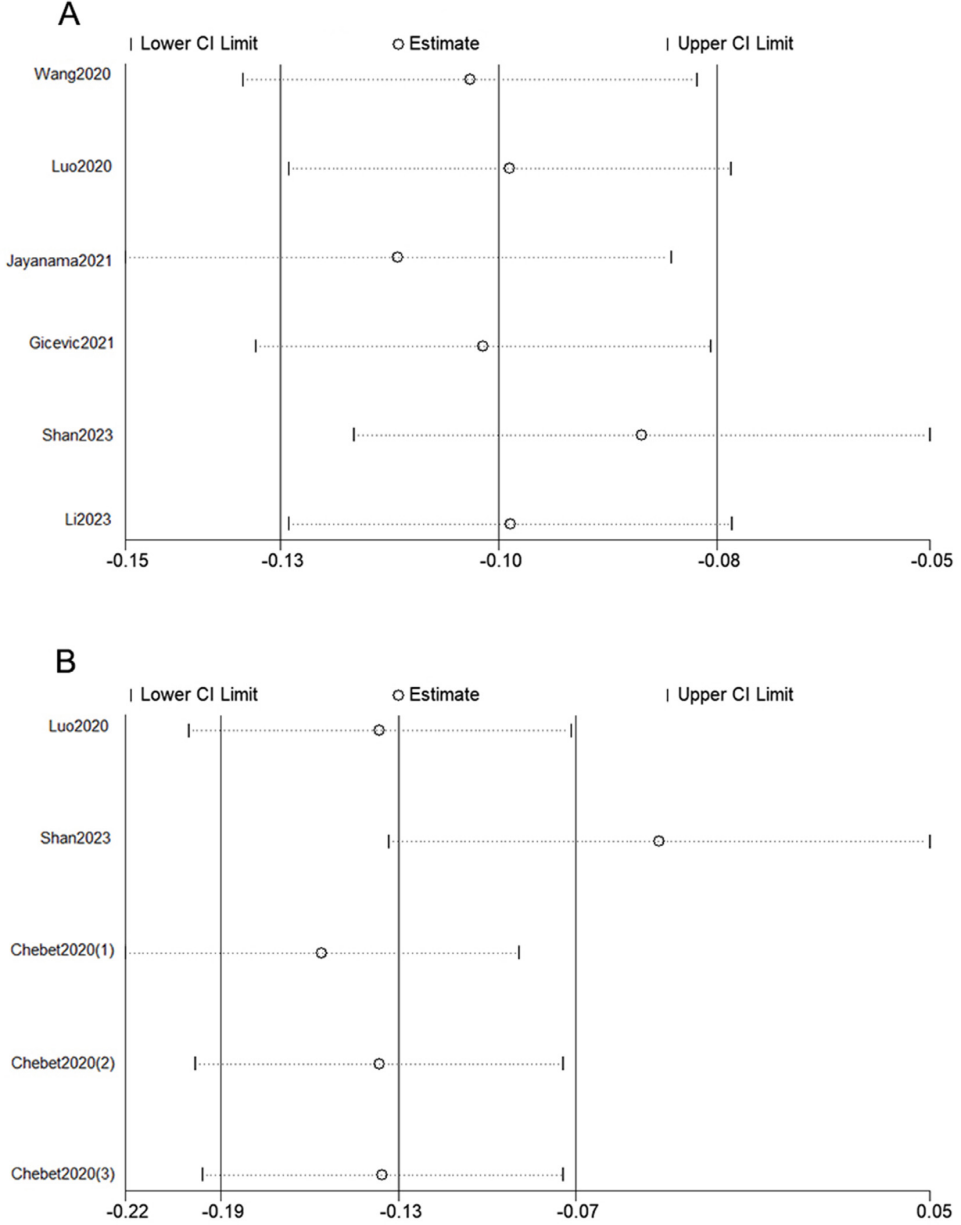
Sensitivity analysis of included studies for the continuous HEI-2015. (A) All-cause mortality. (B) Cancer-cause mortality. HEI, Healthy Eating Index.

Subgroup analysis was performed based on region, sex, sample size, and duration of follow-up as shown in Table 2. The pooled HRs illustrated that compared with the lowest HEI category, the highest HEI category was associated with a lower estimated risk of all-cause and cause-specific mortality in all subgroups except for the subgroup with sample size <15,000 participants for cancer-cause mortality (HR: 0.89; 95% CI: 0.71, 1.07; *I*^2^: 0%; *P* = 0.793). As presented in Supplemental Figure 1, for all-cause mortality, heterogeneity was found when the sample size was >15,000 participants (*I*^2^: 62.6%; *P* = 0.006), as well as in male group (*I*^2^: 83.8%; *P* < 0.1). No heterogeneity was found in the subgroup analyses for cancer-cause mortality (Supplemental Figure 2). In the analysis of the HEI-2015 and CVD-cause mortality, heterogeneity was observed in both male and female groups (*I*^2^: 81.3%; *P* = 0.005 and *I*^2^: 57.5%; *P* = 0.07, respectively) as shown in Supplemental Figure 3. To measure the effects of each individual study on the pooled HRs, each single study was omitted in sequence each time. The results demonstrated that our results were statistically credible as shown in Figure 5 (the HEI-2015 as categorical variable) and Figure 6 (the HEI-2015 as continuous variable).

**TABLE 2:** Combined results of subgroup analysis for HEI-2015 and risk of mortality (highest HEI-2015 vs. the lowest category)

Outcome	Stratification criterion	Number of included studies (*n*)	Pooled HR (95% CI)	Heterogeneity	
				*I*^2^ (%)	*P* value∗
All-cause mortality	Gender				
	Male	5	0.84 (0.78, 0.89)	83.8	<0.001
	Female	8	0.78 (0.76,0.80)	20.4	0.268
	Region				
	United States	13	0.80 (0.79, 0.81)	24.4	0.197
	Non-United States	3	0.90 (0.82, 0.98)	0	0.613
	Sample size				
	<15,000	8	0.82 (0.77, 0.87)	0	0.959
	>15,000	9	0.81 (0.79, 0.83)	62.6	0.006
	Follow-up period				
	<15 y	10	0.83 (0.81, 0.86)	27.9	0.188
	≥15 y	7	0.8 (0.79, 0.81)	0	0.476
Cancer-cause mortality	Gender				
	Male	2	0.79 (0.75, 0.82)	0	0.587
	Female	5	0.82 (0.78, 0.85)	0	0.798
	Region				
	United States	9	0.8 (0.78, 0.83)	0	0.792
	Non-United States	2	0.83 (0.69, 0.97)	0	0.364
	Sample size				
	<15,000	3	0.89 (0.71, 1.07)	0	0.793
	>15,000	9	0.8 (0.78, 0.83)	0	0.772
	Follow-up period				
	<15 y	6	0.85 (0.79, 0.90)	0	0.954
	≥15 y	6	0.8 (0.77, 0.82)	0	0.701
CVD-cause mortality					
	Gender				
	Male	3	0.82 (0.72, 0.94)	81.3	0.005
	Female	4	0.80 (0.73, 0.87)	57.5	0.07

Abbreviations: CI, confidence interval; CVD, cardiovascular disease; HEI, Healthy Eating Index; HR, hazard ratio.

∗ *P* value for heterogeneity within each subgroup.

### Publication bias

Funnel plots (Supplemental Figures 4 and 5) and Egger's test (Table 3) indicated no evidence of significant publication bias whether considering the HEI-2015 as continuous variable (all-cause mortality: *P* = 0.98, cancer-mortality: *P* = 0.088) or categorical variable (all-cause mortality: *P* = 0.24, CVD-mortality: *P* = 0.105, cancer-mortality: *P* = 0.936) detected in this meta-analysis.

**TABLE 3:** Summary of the Egger’s test results for publication bias assessment

		*t* value	*P* value
The highest HEI-2015 vs. the owest category HEI-2015	All-cause mortality	1.22	0.24
	Cancer-cause mortality	1.78	0.105
	CVD-cause mortality	−0.08	0.936
Continuous HEI-2015	All-cause mortality	−0.03	0.98
	Cancer-cause mortality	2.5	0.088

Abbreviations: CVD, cardiovascular disease; HEI, Healthy Eating Index.

## Discussion

In this study, the significant associations between the HEI-2015 and the outcomes of all-cause and cause-specific mortality were indicated based on a broad range of population containing 1,065,175 participants from 20 studies. Moreover, the findings from our study indicated that compared with the lower HEI-2015, the higher HEI-2015 was related to a lower risk of all-cause mortality with a nonlinear dose–response relationship, which observed that an increment of each score in the HEI-2015 was associated with lower risk of mortality from all-cause. Furthermore, a linear relationship was found between the HEI-2015 and cancer-cause, as well as CVD-cause mortality, which showed a decreasing trend of cancer-cause and CVD-cause mortality for each 1 unit increase in HEI-2015 score. To the best of our knowledge, this is the first dose–response meta-analysis to provide a comprehensive assessment to the relationship between the HEI-2015 and all-cause, CVD-cause, and cancer-cause mortality, which supports that the DGA recommendations to improve dietary quality might lead to a longer life expectancy.

Dietary habits are crucial in maintaining health. According to the Global Burden of Disease Study 2017, suboptimal diet acts as a leading cause of mortality because it is responsible for 1 in every 5 deaths across the globe [39]. As a preventable risk factor, the improvement of human dietary quality could potentially decrease the risk of mortality [16]. Therefore, dietary recommendations based on real-life evidence are essential for people to comply with. When analyzing the relationship between diet and health outcomes, the highlight shifts from focusing on individual nutrients or food to overall dietary patterns [40]. The dietary patterns account for the complexity and intercorrelation of different dietary components because human food intake is always multidimensional and single component analysis may be inadequate [41]. To define and quantify dietary patterns, 2 approaches are introduced as tools: a priori approach and a posteriori approach [42]. The former is based on statistical exploratory methods through dietary intake including factor analysis and cluster analysis, and the latter evaluates the compliance with the current nutrition knowledge such as specific dietary pattern or the recommended dietary guidelines, known as dietary indices, including HEI, Dietary Approaches to Stop Hypertension (DASH), Diet Quality Index, Mediterranean Diet Score (MDS), as well as Dietary Guidelines Index [43].

As a dietary quality measure derived from the DGAs, the HEI is subsequently updated, and has been used to describe diet quality in different populations globally, as well as to evaluate the association between diet quality and health outcomes [7]. A higher HEI-2015 score is indicative of better adherence to the dietary patterns recommended by DGA and related to lower risk of NCDs including CVD, cancer, and diabetes in the general population in previous epidemiological studies [4,[10], [11], [12], [13], [14]]. The HEI-2015 is described as densities instead of individual amounts and the score of each component is obtained by comparing the density with relevant scoring standards [7]. On this account, the HEI-2015 could be used to appraise the diet quality of any mix of foods. Nevertheless, issues should not be ignored considering with the components of the HEI-2015. The sources of information on food composition might differ among the different databases, which could influence the dietary constituent amounts [44]. In addition, the dietary intake data based on such self-report methods as food frequency questionnaires and food records may reduce measurement errors [45]. The results of our analysis revealed that a higher HEI-2015, which could also be considered as better abiding by the DGA, was related to the reduction in risk of all-cause, CVD-cause, and cancer-cause mortality. However, we only explored the relationship between total score and mortality; the correction between the alignment with the recommended intake of the individual HEI-2015 components and mortality was not analyzed. The total score could be obtained through different files of component scores and the components in the HEI-2015 may be related to or interact with one another [21,44]. Further comprehensive analyses for the effect of the multiple separate HEI-2015 components on total score and health outcomes including mortality are still called for to provide stronger evidence for specific dietary recommendations formulation.

A diet with a high HEI-2015 score is considered to be a healthy and balanced dietary pattern, which has a myriad of minerals and vitamins from fruits, vegetables, whole grains, dairy or soy alternatives, protein foods, and unsaturated fatty acids [7]. The possible biological mechanisms underlying a healthy diet’s role in reducing mortality remain unclear; however, some studies have found that healthy diets may decrease systemic inflammation and oxidative stress, two factors which play a critical role in the development of chronic diseases [46,47]. As a critical aspect in the pathophysiology of CVD and cancer, inflammatory status is illustrated to have an association with diet in previous studies [48,49]. Fruits and vegetables are good sources of nutrients with antioxidant capacity, including minerals and vitamins, which contribute to reactive oxygen species detoxification, as well as reduce the disruption of redox control and DNA damage so as to protect the organism from the adverse effects of oxidative stress [47,50]. Higher HEI-2015 component scores for refined grains, sodium, saturated fats, and added sugars are characterized by a lower consumption of these dietary components, which have been linked to outcomes such as obesity, dyslipidemia, or increased incidence of CVD [7,51].

Despite this being the first meta-analysis about the association of HEI-2015 with mortality, some limitations of our study should also be acknowledged when interpreting the results. First, the number of studies included in this meta-analysis was relatively small, which might affect the conclusion, so the results should be cautiously interpreted. Second, significant between-study heterogeneity was observed in our study, especially in the CVD-cause group, which might influence the results of this meta-analysis. Hence, for the sake of producing a relatively conservative estimate, a random-effect model was used. We also conducted the subgroup analyses based on gender, region, sample size, and follow-up years, which revealed that the different effect on all-cause mortality might be attributed to gender and sample size but the variables could not explain the heterogeneity completely. The self- reported data might be prone to misreporting and the recall bias might exist. Furthermore, the HEI-2015 was analyzed as a categorical or continuous variable in different studies; we analyzed them separately for more homogeneous results. However, different categorical cut-off values for the HEI-2015 were chosen in the included studies, some studies used tertiles and some used quartiles or quintiles and these might potentially affect the results. To minimize the impact in this regard, the highest and the lowest category were compared. The heterogeneity may also partly owe to the various baseline characteristics of included studies. However, because the data in our study were extracted from published articles instead of investigating individual patient data, sufficient information for deep layer analysis such as meta-regression and subgroup analyses by other potential confounders was limited. Third, this study is a literature-based analysis; therefore, the possibility of publication bias may exist. However, no publication bias was detected from funnel plots and Egger's test. Fourth, the diet quality was only assessed by the HEI-2015 in this meta-analysis and other indices such as DASH and MDS were not included, which limited the generalizability and generalization of this study. Therefore, further studies with different dietary quality indices are needed to confirm the results.

In conclusion, this systematic review and dose–response meta-analysis is the first one to quantitatively demonstrate the pooled risk of mortality from all-cause, CVD and cancer according to the dietary patterns defined by the HEI-2015. In this study, we found that a higher HEI-2015 was associated with a lower risk of mortality from all-cause, CVD and cancer. Further large prospective studies are still needed to provide more comprehensive information on the potential effects of dietary patterns assessed by the HEI-2015 on the risk of mortality.

### Author contributions

The authors’ responsibilities were as follows – DYL: design and supervision; XYH, DYL: writing and final content; and both authors: read and approved the manuscript.

### Conflict of interest

The authors report no conflicts of interest.

### Funding

The authors reported no funding received for this study.
