# Supplementary material for: The Healthy Eating Index-2015 and All-Cause/Cause-Specific Mortality: A Systematic Review and Dose–Response Meta-Analysis
Source: Adv Nutr. 2024 Feb 23;15(3):100166. doi: 10.1016/j.advnut.2023.100166 (PMC10980904; doi:10.1016/j.advnut.2023.100166)
Supplement: Multimedia component 1 [file mmc1.docx]

**Title: Healthy Eating Index-2015 (HEI-2015) and all-cause/cause-specific mortality: a systematic review and dose-response meta-analysis**

Authors: Xuanyu Hao et al.

**Supplementary Table 1** PRISMA 2009 Checklist.

| **Section/topic** | **#** | **Checklist item** | **Reported on page #** |
| --- | --- | --- | --- |
| **TITLE** | | |  |
| Title | 1 | Identify the report as a systematic review, meta-analysis, or both. | 1 |
| **ABSTRACT** | | |  |
| Structured summary | 2 | Provide a structured summary including, as applicable: background; objectives; data sources; study eligibility criteria, participants, and interventions; study appraisal and synthesis methods; results; limitations; conclusions and implications of key findings; systematic review registration number. | 2 |
| **INTRODUCTION** | | |  |
| Rationale | 3 | Describe the rationale for the review in the context of what is already known. | 3 |
| Objectives | 4 | Provide an explicit statement of questions being addressed with reference to participants, interventions, comparisons, outcomes, and study design (PICOS). | 3 |
| **METHODS** | | |  |
| Protocol and registration | 5 | Indicate if a review protocol exists, if and where it can be accessed (e.g., Web address), and, if available, provide registration information including registration number. | 4 |
| Eligibility criteria | 6 | Specify study characteristics (e.g., PICOS, length of follow-up) and report characteristics (e.g., years considered, language, publication status) used as criteria for eligibility, giving rationale. | 4 |
| Information sources | 7 | Describe all information sources (e.g., databases with dates of coverage, contact with study authors to identify additional studies) in the search and date last searched. | 3-4 |
| Search | 8 | Present full electronic search strategy for at least one database, including any limits used, such that it could be repeated. | 3-4 |
| Study selection | 9 | State the process for selecting studies (i.e., screening, eligibility, included in systematic review, and, if applicable, included in the meta-analysis). | 4 |
| Data collection process | 10 | Describe method of data extraction from reports (e.g., piloted forms, independently, in duplicate) and any processes for obtaining and confirming data from investigators. | 4 |
| Data items | 11 | List and define all variables for which data were sought (e.g., PICOS, funding sources) and any assumptions and simplifications made. | 4 |
| Risk of bias in individual studies | 12 | Describe methods used for assessing risk of bias of individual studies (including specification of whether this was done at the study or outcome level), and how this information is to be used in any data synthesis. | 4 |
| Summary measures | 13 | State the principal summary measures (e.g., risk ratio, difference in means). | 4-5 |
| Synthesis of results | 14 | Describe the methods of handling data and combining results of studies, if done, including measures of consistency (e.g., I^2^) for each meta-analysis. | 4-5 |

Page 1 of 2

| **Section/topic** | **#** | **Checklist item** | **Reported on page #** |
| --- | --- | --- | --- |
| Risk of bias across studies | 15 | Specify any assessment of risk of bias that may affect the cumulative evidence (e.g., publication bias, selective reporting within studies). | 4 |
| Additional analyses | 16 | Describe methods of additional analyses (e.g., sensitivity or subgroup analyses, meta-regression), if done, indicating which were pre-specified. | 4-5 |
| **RESULTS** | | |  |
| Study selection | 17 | Give numbers of studies screened, assessed for eligibility, and included in the review, with reasons for exclusions at each stage, ideally with a flow diagram. | 5 |
| Study characteristics | 18 | For each study, present characteristics for which data were extracted (e.g., study size, PICOS, follow-up period) and provide the citations. | 5-6 |
| Risk of bias within studies | 19 | Present data on risk of bias of each study and, if available, any outcome level assessment (see item 12). | 6 |
| Results of individual studies | 20 | For all outcomes considered (benefits or harms), present, for each study: (a) simple summary data for each intervention group (b) effect estimates and confidence intervals, ideally with a forest plot. | 6-7 |
| Synthesis of results | 21 | Present results of each meta-analysis done, including confidence intervals and measures of consistency. | 6-7 |
| Risk of bias across studies | 22 | Present results of any assessment of risk of bias across studies (see Item 15). | 7 |
| Additional analysis | 23 | Give results of additional analyses, if done (e.g., sensitivity or subgroup analyses, meta-regression [see Item 16]). | 7 |
| **DISCUSSION** | | |  |
| Summary of evidence | 24 | Summarize the main findings including the strength of evidence for each main outcome; consider their relevance to key groups (e.g., healthcare providers, users, and policy makers). | 8-9 |
| Limitations | 25 | Discuss limitations at study and outcome level (e.g., risk of bias), and at review-level (e.g., incomplete retrieval of identified research, reporting bias). | 9-10 |
| Conclusions | 26 | Provide a general interpretation of the results in the context of other evidence, and implications for future research. | 10 |
| **FUNDING** | | |  |
| Funding | 27 | Describe sources of funding for the systematic review and other support (e.g., supply of data); role of funders for the systematic review. | None |

*From:*  Moher D, Liberati A, Tetzlaff J, Altman DG, The PRISMA Group (2009). Preferred Reporting Items for Systematic Reviews and Meta-Analyses: The PRISMA Statement.

For more information, visit: [**www.prisma-statement.org**](http://www.prisma-statement.org).

| Supplemental Table 2 Details of bias assessment in each domain and overall risk of bias with ROBINS-E tool. | | | | | | | | |
| --- | --- | --- | --- | --- | --- | --- | --- | --- |
| Studies | Risk of bias due to confounding | Risk of bias arising from measurement of the exposure | Risk of bias in selection of participants into the study (or into the analysis) | Risk of bias due to post-exposure interventions | Risk of bias due to missing data | Risk of bias arising from measurement of the outcome | Risk of bias in selection of the reported result | Overall risk of bias |
| Myneni et al.  2021 | High risk | High risk | Low risk | Low risk | Some concerns | Low risk | Some concerns | High risk |
| Panizza et al.  2018 | High risk | High risk | Low risk | Low risk | Low risk | Low risk | Some concerns | High risk |
| Haslam et al.  2023 | High risk | High risk | Low risk | Low risk | Some concerns | Low risk | Some concerns | High risk |
| Hu et al.  2019 | High risk | High risk | Low risk | Low risk | Low risk | Low risk | Some concerns | High risk |
| Hu et al.  2020 | Some concerns | High risk | Low risk | Low risk | Low risk | Low risk | Some concerns | High risk |
| Wang et al.  2020 | High risk | High risk | Low risk | Low risk | Some concerns | Low risk | Some concerns | High risk |
| Reedy et al.  2018 | High risk | High risk | Low risk | Low risk | Some concerns | Low risk | Some concerns | High risk |
| Chebet et al.  2020 | High risk | High risk | Low risk | Low risk | Some concerns | Low risk | Some concerns | High risk |
| Jayanama et al.  2021 | High risk | High risk | Low risk | Low risk | Some concerns | Low risk | Some concerns | High risk |
| Ha et al.  2020 | High risk | High risk | Low risk | Low risk | Low risk | Low risk | Some concerns | High risk |
| Li et al.  2023 | Some concerns | High risk | Low risk | Low risk | Low risk | Low risk | Some concerns | High risk |
| Luo et al.  2020 | High risk | High risk | Low risk | Low risk | Some concerns | Low risk | Some concerns | High risk |
| Hashemian et al.  2019 | High risk | High risk | Low risk | Low risk | Low risk | Low risk | Some concerns | High risk |
| Lopez-Pentecost et al.  2022 | High risk | High risk | Low risk | Low risk | Some concerns | Low risk | Some concerns | High risk |
| Gicevic et al.  2021 | High risk | High risk | Low risk | Low risk | Some concerns | Low risk | Some concerns | High risk |
| Park et al.  2022 | Some concerns | High risk | Low risk | Low risk | Some concerns | Low risk | Some concerns | High risk |
| George et al.  2020 | Some concerns | High risk | Low risk | Low risk | Some concerns | Low risk | Some concerns | High risk |
| Shan et al.  2023 | High risk | High risk | Low risk | Low risk | Some concerns | Low risk | Some concerns | High risk |
| Li Fang et al.  2023 | High risk | High risk | Low risk | Low risk | Low risk | Low risk | Some concerns | High risk |
| Wang et al.  2023 | High risk | High risk | Low risk | Low risk | Low risk | Low risk | Some concerns | High risk |

**
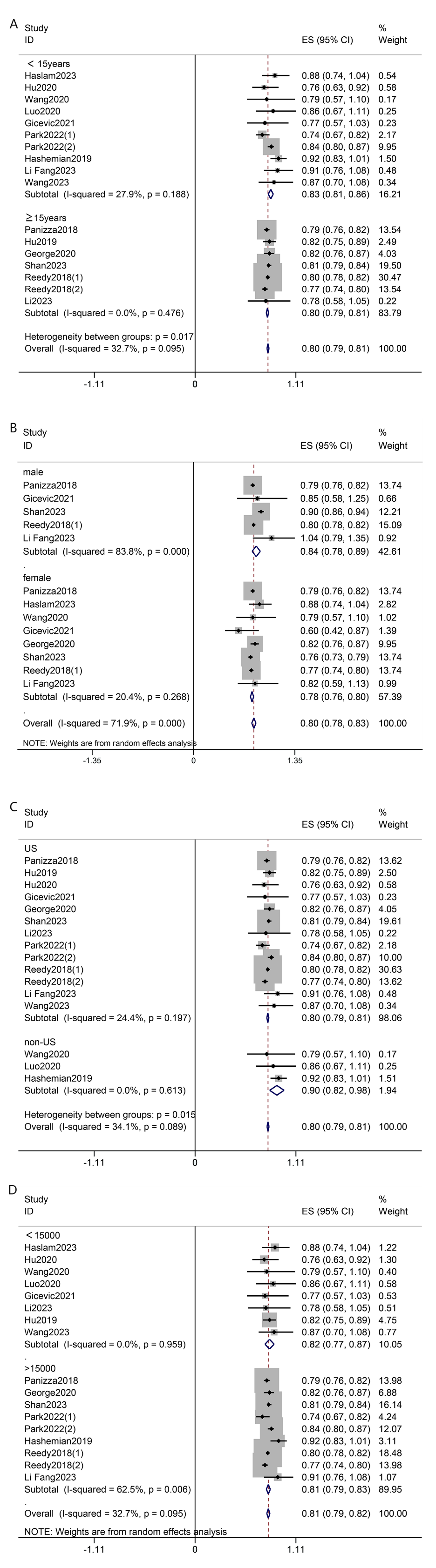
Supplementary Figure 1** Subgroup analyses of included studies for HEI-2015 and risk of all-cause mortality (highest HEI-2015 versus the lowest category) based on (A) duration of follow-up, (B) gender, (C) region, and (D) sample size.


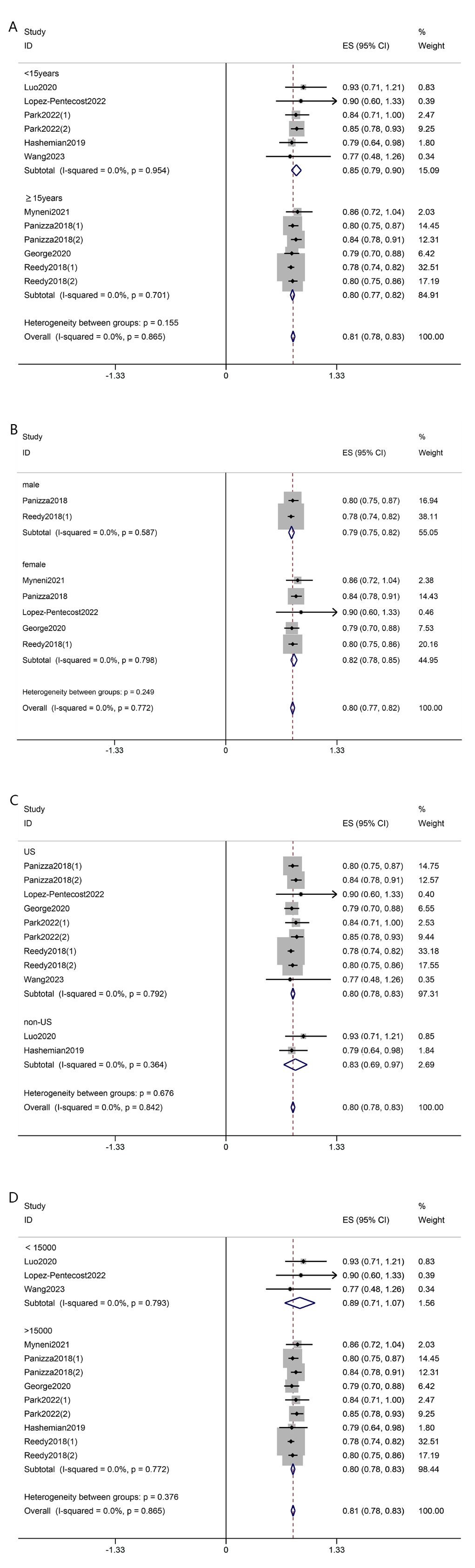
**Supplementary Figure 2** Subgroup analyses of included studies for HEI-2015 and risk of cancer-cause mortality (highest HEI-2015 versus the lowest category) based on (A) duration of follow-up, (B) gender, (C) region, and (D) sample size.


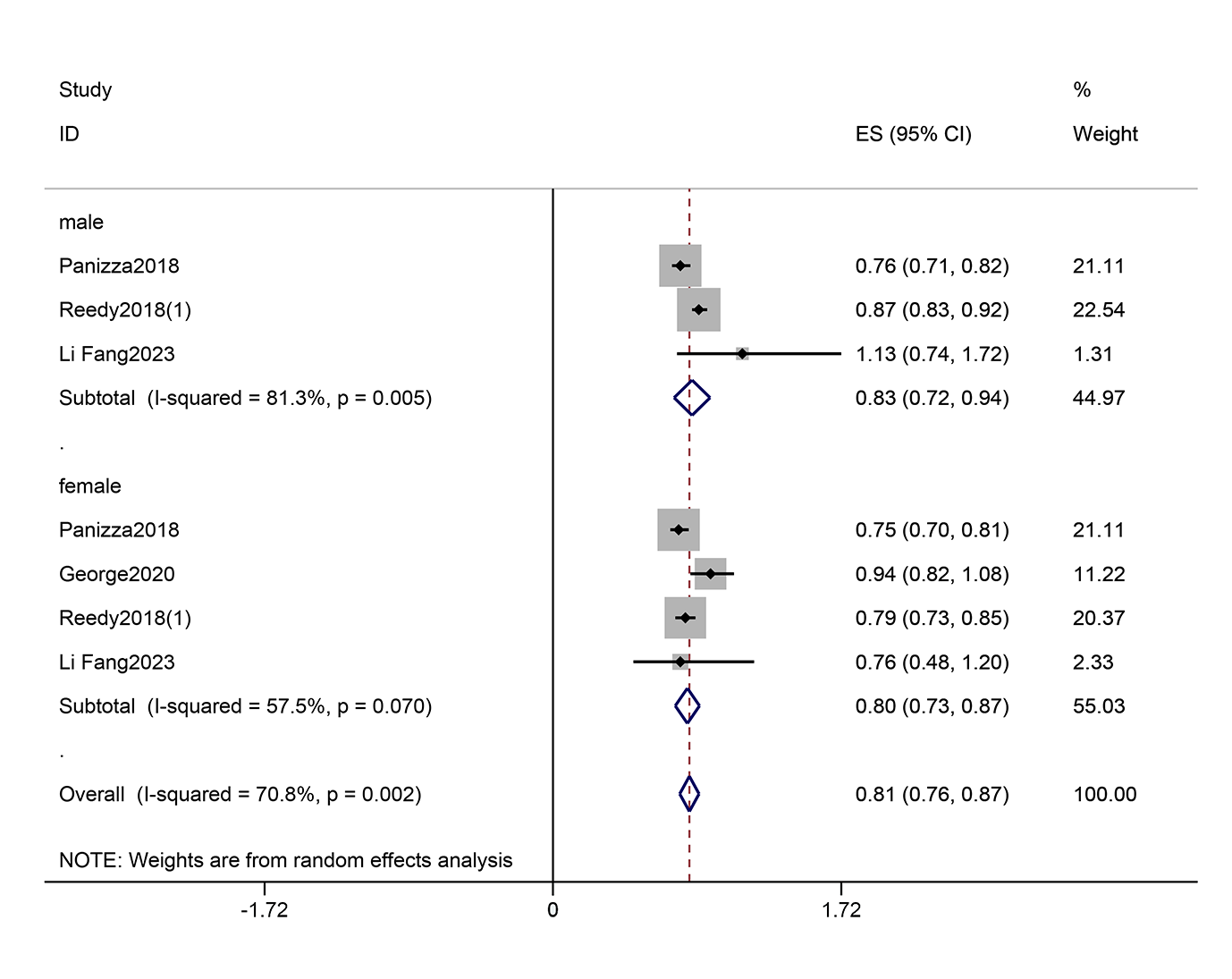
**Supplementary Figure 3** Subgroup analyses of included studies for HEI-2015 and risk of CVD-cause mortality (highest HEI-2015 versus the lowest category) based on gender.


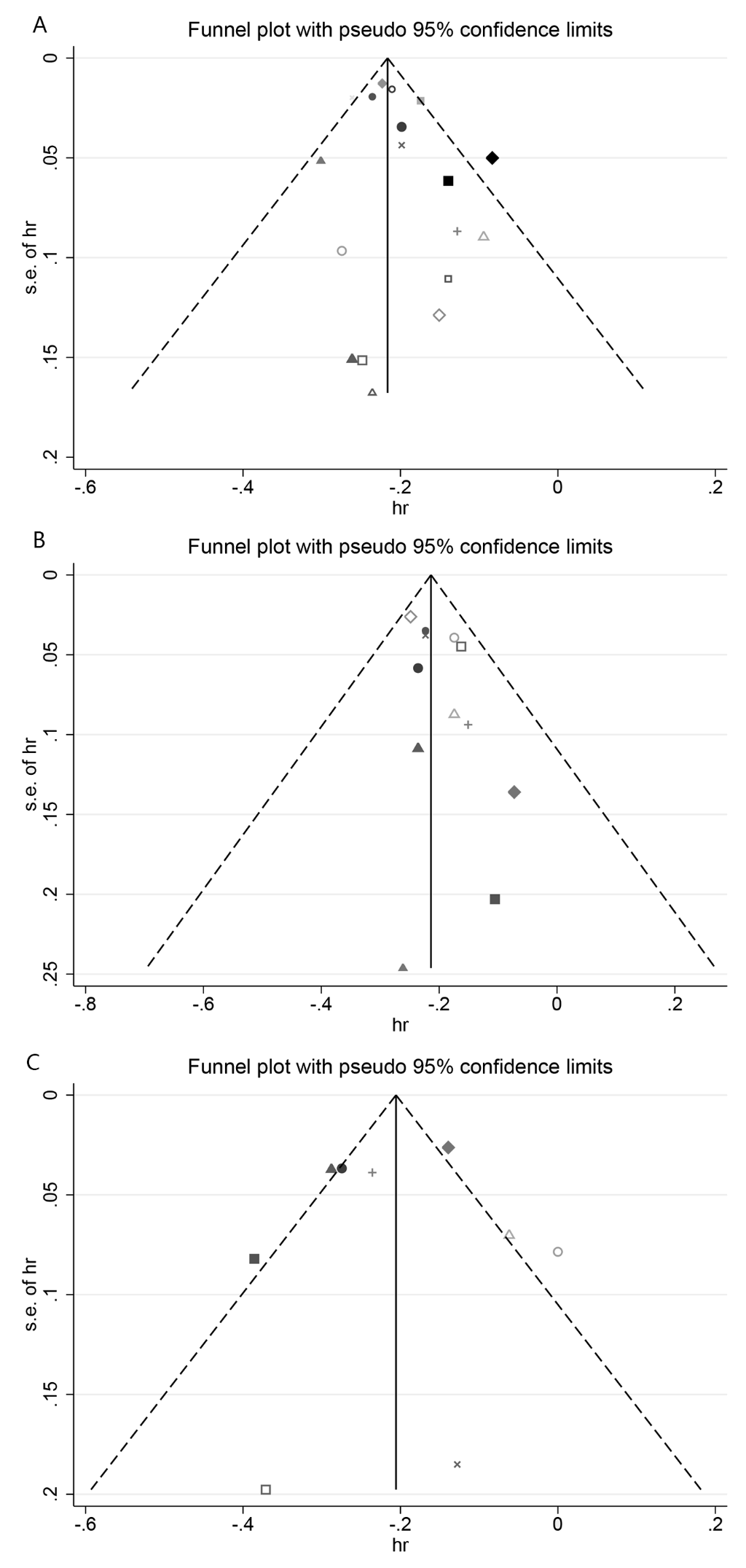
**Supplementary Figure 4** Funnel plot of the included studies for the highest HEI-2015 versus the lowest category HEI-2015. (A) All-cause mortality. (B) Cancer-cause mortality. (C) CVD-cause mortality.


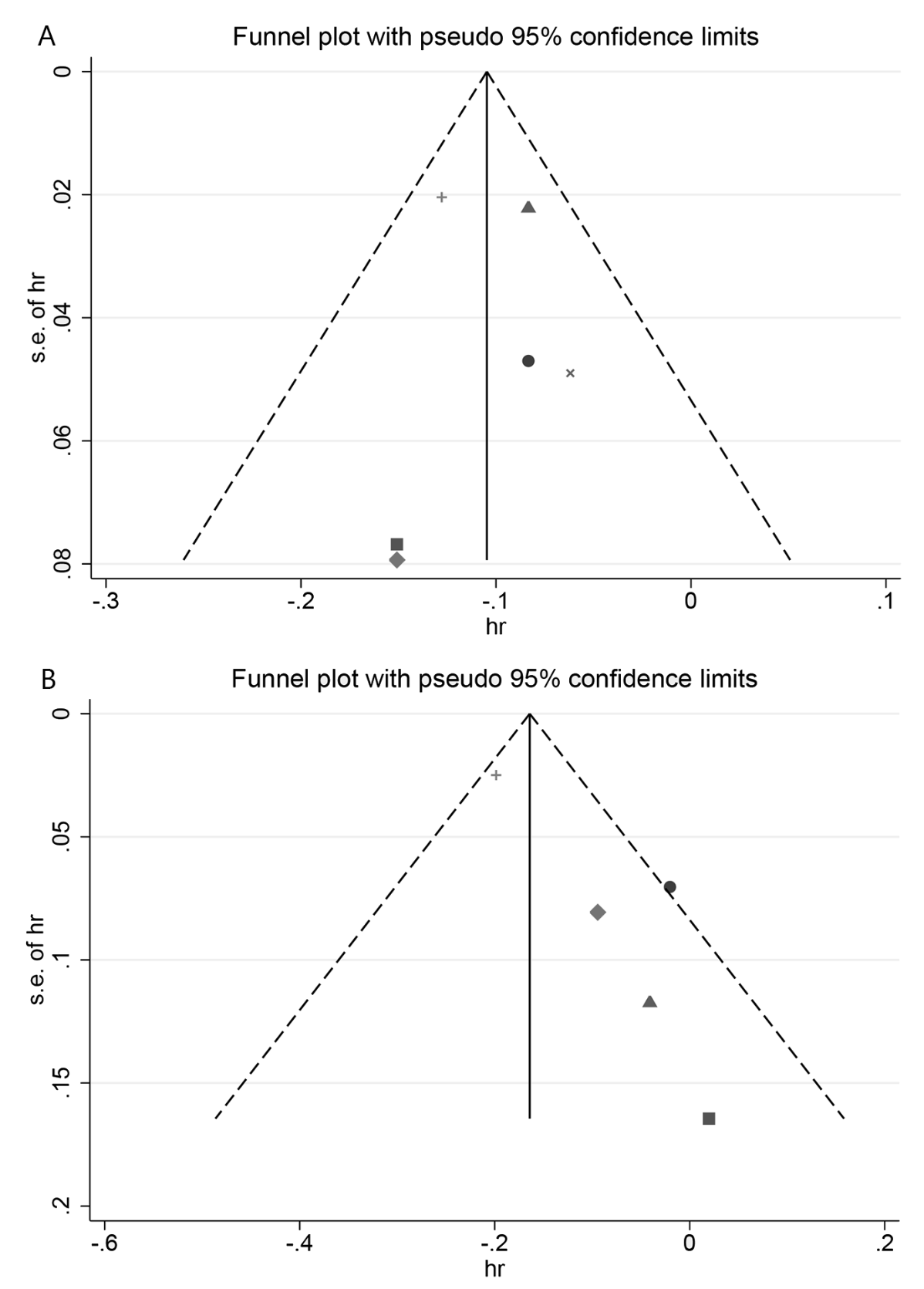
**Supplementary Figure 5** Funnel plot of the included studies for the continuous HEI-2015. (A) All-cause mortality. (B) Cancer-cause mortality.
